# Usefulness of Platelet Distribution Width and Fibrinogen in Predicting In-stent Restenosis With Stable Angina and Type 2 Patients With Diabetes Mellitus

**DOI:** 10.3389/fcvm.2022.710804

**Published:** 2022-03-21

**Authors:** Dayang Chai, Xi Yang, Aichao Wang, Shu Lu, Yuxiang Dai, Jing Zhou

**Affiliations:** ^1^Department of Cardiology, The First People's Hospital of Taicang, The Affiliated Taicang Hospital of Soochow University, Taicang, China; ^2^Department of Endocrinology, The First People's Hospital of Taicang, The Affiliated Taicang Hospital of Soochow University, Taicang, China; ^3^Department of Cardiology, Zhongshan Hospital Affiliated of Fudan University, Shanghai, China

**Keywords:** platelet distribution width (PDW), fibrinogen, stable angina pectoris (SAP), type 2 diabetes mellitus, in-stent restenosis, drug-eluting stent implantation (DES-PCI), inflammation

## Abstract

**Aim:**

The purpose of this study was to investigate the predicting value of platelet distribution width (PDW) and fibrinogen for in-stent restenosis (ISR) in patients with stable angina pectoris and type 2 diabetes mellitus (T2DM) after drug-eluting stent (DES) implantation.

**Methods:**

We enrolled 161 patients who were readmitted with recurrent chest pain and successfully reviewed for coronary angiography and were divided into the ISR and non-ISR groups. We compared the levels of PDW and fibrinogen between the two groups. Logistic regression was used for analyzing independent predictors of ISR. The receiver operating characteristic (ROC) curve analysis was used to determine the optimum cutoff points of PDW and fibrinogen to predict ISR. The Kaplan–Meier survival curves for target lesion failure (TLF) by levels of PDW and fibrinogen.

**Results:**

The multivariate logistic regression analysis showed that PDW and fibrinogen were independent predictors of ISR [odds ratio (OR) = 1.209, 95% CI: 1.024–1.427, *p* = 0.025; OR = 1.006, 95% CI: 1.002–1.011, *p* = 0.010, respectively]. The ROC analyses showed that PDW ≥ 13.15% and fibrinogen ≥ 333.5 mg/dl were predictive of ISR in patients with stable angina pectoris and T2DM after DES implantation. However, the Kaplan–Meier estimate for TLF showed no statistical significance.

**Conclusion:**

Higher levels of PDW and fibrinogen were associated with the incidence of ISR in patients with stable angina with T2DM after DES implantation, but were not independent predictors of TLF.

## Introduction

Although drug-eluting stents (DESs) have significantly reduced the incidence of in-stent restenosis (ISR), ISR remains one of the major complications after DES implantation (1, 2). Elevated fibrinogen level is a recognized risk factor for adverse cardiovascular events in healthy subjects (3–6) and in patients with coronary or peripheral artery disease (7–9). Previous studies have shown that elevated fibrinogen levels predict restenosis in patients with stable atherosclerotic treated with peripheral angioplasty (10, 11). Recently, study showed that the fibrinogen albumin ratio is significantly associated with the development of ISR in patients with coronary artery disease undergoing DES implantation (12). Currently, there is a high rate of ISR in patients with diabetes after DES implantation, and it is important to have all the new factors that can predict ISR in patients with diabetes. However, the relationship between fibrinogen levels and ISR in patients with stable angina pectoris and type 2 diabetes mellitus (T2DM) after DES implantation is unclear.

Platelets play an important role in ISR and neointimal hyperplasia (13). Platelet distribution width (PDW) is simple platelet parameters that are increased during platelet activation. PDW is considered to be a more specific marker of platelet activation because it does not increase during simple platelet swelling (14, 15). PDW has been introduced as a marker of inflammation besides its role in platelet functions. Recent studies found a significant association between PDW and various conditions including diabetic nephropathy, irritable bowel disease, autoimmune hepatitis, and coronary artery disease (16–19). A recent study showed that PDW is an independent predictor of ISR after DES implantation in patients with coronary artery disease and T2DM (20). However, patients with coronary artery disease, including angina pectoris and myocardial infarction, were included in this study. Whether all the PDW has predictive value on the occurrence of ISR after DES implantation in patients with stable angina pectoris is still unknown.

The aim of this study was to evaluate the predicting value of PDW and fibrinogen for ISR in patients with stable angina pectoris and T2DM.

## Materials and Methods

### Study Population and Design

This was the two-center retrospective observational study. We screened a total of 452 patients with stable angina with T2DM, of whom 271 patients underwent DES implantation successfully from January 2016 to January 2019 in our hospitals. All the enrolled patients were followed-up and 161 patients were readmitted with recurrent chest pain and successfully reviewed for coronary artery angiography. According to the results of quantitative coronary angiography (QCA) from follow-up coronary artery angiography, the patients were divided into the ISR (*n* = 27) and non-ISR (*n* = 134) groups. ISR was defined as restenosis ≥ 50% inside the stent or 5 mm proximally or distally in the target vessel. Target lesion failure (TLF) was defined as ISR ≥ 70% with clinical symptoms. It includes criteria: age ≥ 18 years old, diagnosis of stable angina pectoris (21), T2DM (22), DES was successfully implanted, and no serious complications occurred during the perioperative; it excludes criteria were as follow: previous coronary artery bypass grafting (CABG), moderate-to-severe heart failure [the New York Heart Association (NYHA) III-IV], long-term oral anticoagulants, severe liver or renal failure (glomerular filtration rate < 30 ml/min/1.73 m^2^), acute or chronic inflammatory diseases, hematological and immune diseases, and malignant tumors.

### Laboratory Data

All the patients were tested for the standard blood workup, liver function, renal function, blood lipid levels, fasting blood glucose, and coagulation examination before the DES implantation as a routine test at our center. The patients were readmitted and underwent coronary angiography successfully for recurrently chest pain, whose laboratory data were extracted from Hospital Information System (HIS) according to the patient's identification number.

### Angiographic Assessment and Follow-Up

The patients successfully completed the coronary artery angiography or percutaneous coronary intervention (PCI) procedure, which was performed in accordance with current guidelines, during the period of hospitalization. Heparin was used during the procedure for an activated clotting time > 250 s. If the procedure lasted more than an hour, an additional 2,000 IU of heparin was administered. Prior to admission, all the patients were advised to take aspirin (300 mg) and clopidogrel (300 mg)/ticagrelor (180 mg) at least 2 h before the PCI. Stent type [Rapamycin (Siromos or Zotamus)] was chosen by the operator during the operation.

Angiotensin-converting enzyme inhibitors (ACEIs), angiotensin receptor blockers (ARBs), β-blockers, calcium channel antagonists [calcium channel blocker (CCB)], statins, and other medications were taken based on patient's actual condition. Patients who met the inclusion criteria after the first successful DES implantation were followed-up for 5 years. All the patients follow-up coronary artery angiography were readmitted for recurrent chest pain after DES implantation. The degree of coronary stenosis was estimated by QCA. All the patients were divided into the ISR group and the non-ISR group according to results of QCA from follow-up coronary artery angiography. Follow-up data were obtained from the hospital archive, the patients, and relatives of the patients.

### Statistical Analysis

Quantitative continuous variables were expressed as mean ± SD, while data not in normal distribution were reported as medians (P25, P75) and compared using the Mann–Whitney *U*-test. Normally distributed continuous variables were compared using the independent-samples *t*-test. Categorical variables were compared by the chi-squared test or Fisher's exact test. The univariate and the multivariate logistic regression analyses were performed including parameters that differed significantly between the groups in order to identify the predictor for ISR. The receiver operating characteristic (ROC) curve analysis was used to determine the optimum cutoff points of PDW and fibrinogen in predicting ISR. The cumulative event rate was estimated from the Kaplan–Meier curves and compared using the log-rank test. A *p*-value of < 0.05 was considered as statistically significant. Statistical analyses were performed using software package SPSS software version 23.0 (SPSS Institute Incorporation, Chicago, Illinois, USA) and GraphPad Prism version 8 (GraphPad Software Incorporation, USA).

## Results

**Figure 1:**
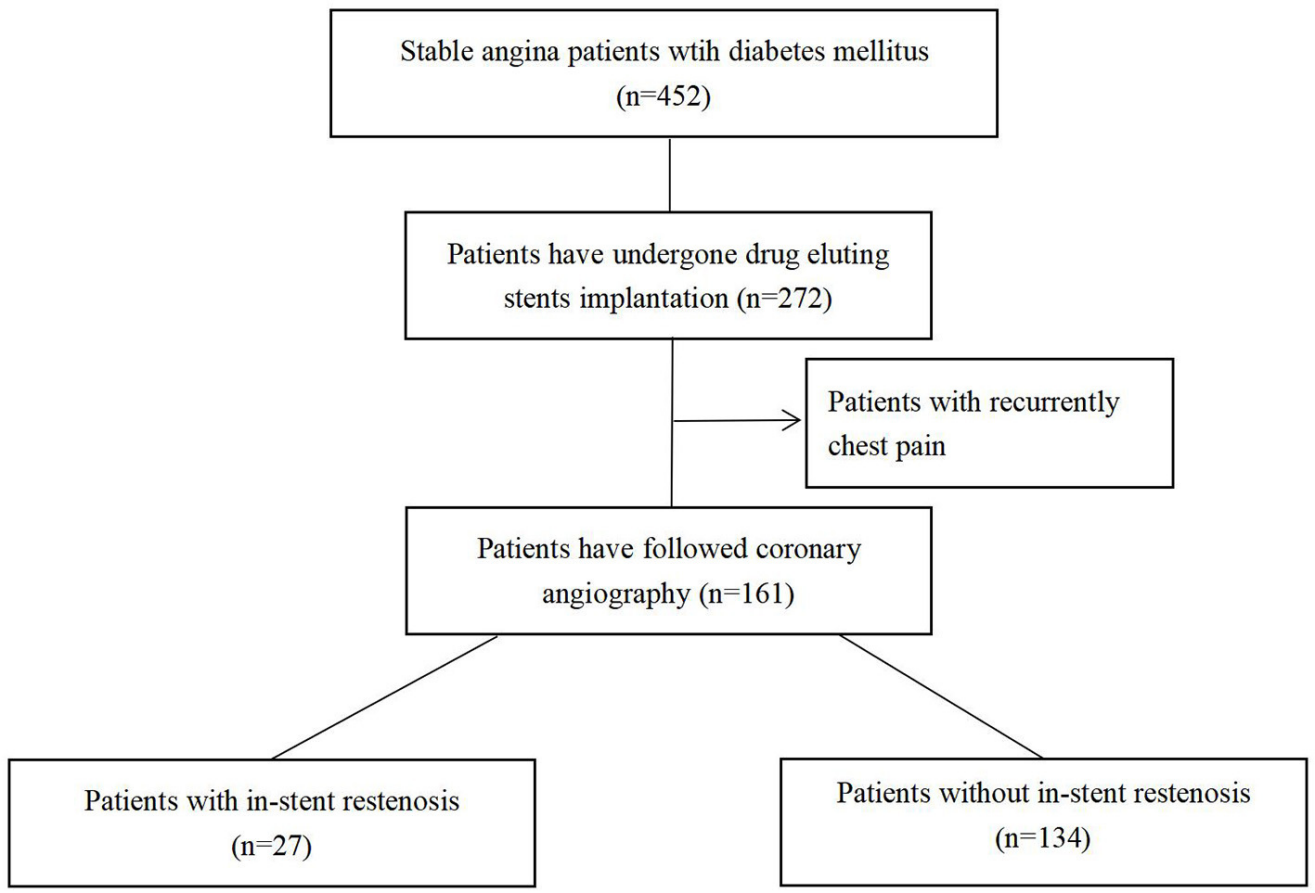
Study flowchart. A total of 161 patients finally enrolled in our study.

Among 272 patients with stable angina with diabetes who successfully completed DES implantation, 161 patients met the inclusion criteria and were, therefore, included in this study. According to the results of followed coronary artery angiography, the incidence of ISR was 16.8% in recurrent patients with chest pain and all the patients were divided into the non-ISR group (*n* = 134) and the ISR group (*n* = 27) (Figure 1).

Baseline clinical characteristics were shown in Table 1. There were no significant differences in age, sex, age, current smoker, hypertension, and prior coronary artery diseases between the ISR and non-ISR groups (all *p* > 0.05). There were no significant differences in total cholesterol, triglyceride, high-density lipoprotein cholesterol (HDL-C), low-density lipoprotein cholesterol (LDL-C), very-low-density lipoprotein cholesterol (VLDL-C), neutrophily/lymphocyte ratio (NLR), monocyte/lymphocyte ratio (MLR), platelet/lymphocyte ratio (PLR) and red cell distribution width (RDW) between the two groups (*p* > 0.05 for all). There was no significant difference in the current oral administration of ACEI, ARB, and other drugs between the two groups. Fibrinogen and PDW levels were higher in patients with ISR than in patients with non-ISR (*p* = 0.046, 0.014, respectively).

**Table 1:** Baseline clinical characteristics.

	**ISR (*n* = 27)**	**Non-ISR (*n* = 134)**	***P*-value**
Age, yrs	64 [64–64]	65 [58–72]	0.564
Male	17 (63.0)	99 (73.9)	0.249
Current smoker	7 (25.9)	61 (45.5)	0.060
Hypertension	23 (85.2)	104 (77.6)	0.379
Dyslipidemia	6 (22.2)	12 (9.0)	0.046
Prior coronary artery diseases	0 (0)	1 (0.7)	-
**Laboratory data**			
HbA1c (%)	7.76 ± 1.57	7.31 ± 1.38	0.137
Total Cholesterol (mmol/L)	3.72 ± 1.00	3.48 ± 0.83	0.192
Triglyceride (mmol/L)	1.93 ± 1.14	1.94 ± 0.62	0.966
LDL-C (mmol/L)	1.91 ± 0.87	1.64 ± 0.73	0.090
HDL-C (mmol/L)	1.05 ± 0.43	1.08 ± 0.34	0.659
VLDL-C (mmol/L)	0.83 ± 0.48	0.76 ± 0.47	0.505
Hemoglobin (g/l)	127.74 ± 19.61	134.25 ± 18.78	0.105
Platelet count (10^9^/*L*)	214.19 ± 78.76	189.51 ± 51.95	0.129
Fibrinogen (mg/dL)	291.00 [240.00–337.25]	266.00 [230.00–309.25]	0.045
RDW (%)	12.97 ± 1.27	12.68 ± 0.82	0.124
PDW (%)	14.68 ± 3.16	13.35 ± 2.38	0.014
NLR	2.43 [1.77–2.83]	2.18 [1.68–2.87]	0.340
MLR	0.287 [0.227–0.358]	0.284 [0.225–0.355]	0.812
PLR	100.69 [82.97–131.33]	119.50 [75.71–140.00]	0.305
**Medications**			
Aspirin	25 (92.6)	127 (94.8)	0.647
Ticagrelor	6 (22.2)	27 (20.1)	0.808
ACEI/ARB	10 (37.0)	50 (37.3)	0.978
β-blockers	9 (33.3)	69 (51.5)	0.085

*Data are presented as N (%), mean ± (SD) and median interquartile range in parentheses*.

*ACEI, angiotensin-converting enzyme inhibitors; ARB, angiotensin receptor blocker; HbA1c, Hemoglobin A1c; HDL-C, high-density lipoprotein cholesterol; ISR, in-stent restenosis; LDL-C, low-density lipoprotein cholesterol; MLR, monocyte/lymphocyte ratio; NLR, neutrophily/lymphocyte ratio; non-ISR, non in-stent restenosis; PDW, platelet distribution width; PLR, platelet/lymphocyte ratio; RDW, red cell distribution width; VLDL-C, very low-density lipoprotein cholesterol*.

Baseline angiographic characteristics for the study population are shown in Table 2. The minimum stent diameter in the ISR group was significantly smaller than that in the non-ISR group (2.70 ± 0.34 vs. 2.87 ± 0.48, *p* = 0.041). There were no significant differences between the two groups in-stent overlap, number of stents, lesion characteristics, and target vessel.

**Table 2:** Angiographic and procedural characteristics of treated lesions.

	**ISR (*n* = 27)**	**Non-ISR (*n* = 134)**	***P*-value**
Number of stents per target vessel	1.41 ± 0.57	1.51 ± 0.69	0.260
Stent length (mm)	39.93 ± 16.7	43.26 ± 23.01	0.382
Stents overlapped	10 (37.0)	58 (43.3)	0.549
Minimum stent diameter (mm)	2.70 ± 0.34	2.87 ± 0.48	0.041
Non-compliant balloon expansion	25 (92.6)	118 (88.1)	0.728
**Complexity of lesions**			
Complex Bifurcation lesions	2 (7.4)	14 (10.4)	0.897
Chronic total occlusion	5 (18.5)	31 (23.1)	0.599
Ostial lesions	6 (22)	21 (15.7)	0.406
**Target coronary artery**			
Left main trunk	1 (3.7)	9 (6.7)	0.817
Left anterior descending artery	14 (51.9)	67 (50.0)	0.861
Left circumflex artery	3 (11.1)	23 (17.2)	0.622
Right coronary artery	10 (37)	44 (32.8)	0.673

*Data are presented as N (%) and mean ± (SD)*.

**Figure 2:**
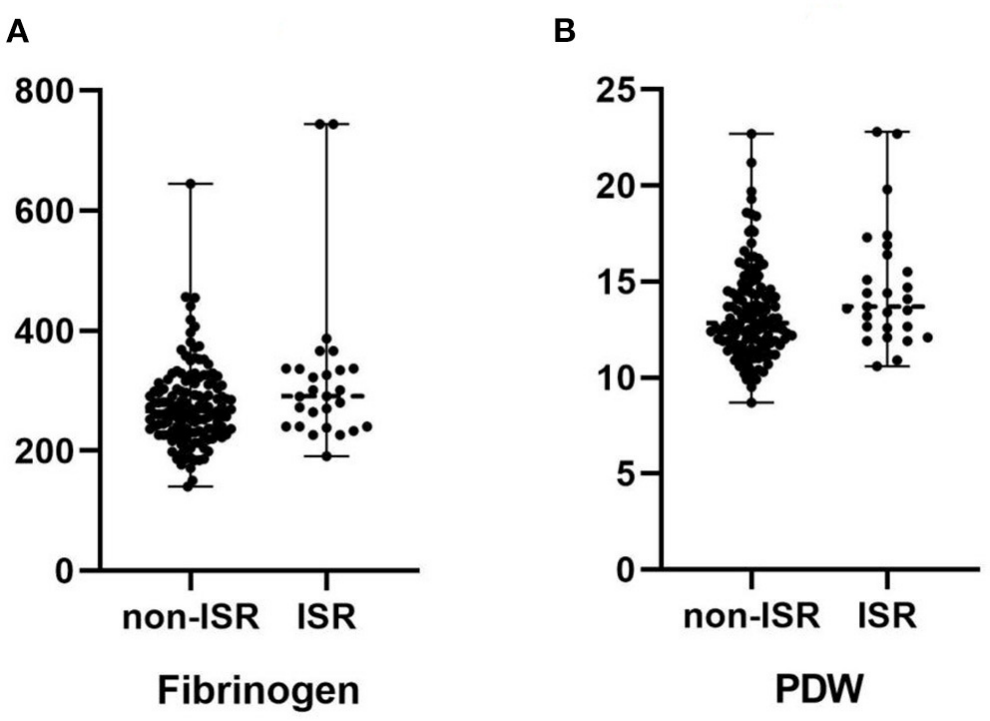
**(A)** Comparison of the median fibrinogen in patients with and without in-stent restenosis. **(B)** Comparison of the median platelet distribution width (PDW) in patients with and without in-stent restenosis.

As shown in Table 3, the univariate logistic regression analysis showed that the PDW, fibrinogen, and LDL-C were statistically significant risk factors for ISR. The multivariate logistic regression analysis showed that fibrinogen and PDW were significantly correlated with ISR (*p* = 0.025, 0.010, respectively) (Figure 2).

**Table 3:** The univariate and multivariate logistic regression analysis for predictors of in-stent restenosis (ISR) for patients in prepercutaneous coronary intervention (PCI).

**Variables**	**Univariable analysis**			**Multivariable analysis**		
	**OR**	**95%CI**	***P*-value**	**OR**	**95%CI**	***P*-value**
Fibrinogen (mg/dL)	1.005	1.001–1.010	0.017	1.006	1.002–1.011	0.010
PDW (%)	1.263	1.057–1.510	0.010	1.209	1.024–1.427	0.025
Current smoker	0.419	0.166–1.057	0.065	0.366	0.131–1.021	0.055
Dyslipidemia	0.344	0.116–1.018	0.054	2.181	0.448–10.63	0.334
LDL-C (mmol/L)	3.011	2.032–4.463	0.013	0.391	0.147–1.041	0.391
Minimum stent diameter (mm)	0.410	0.143–1.174	0.097	0.612	0.191–1.960	0.409

*CI, confidence interval; LDL-C, low-density lipoprotein cholesterol; OR, odds ratio; PDW, platelet distribution width*.

**Figure 3:**
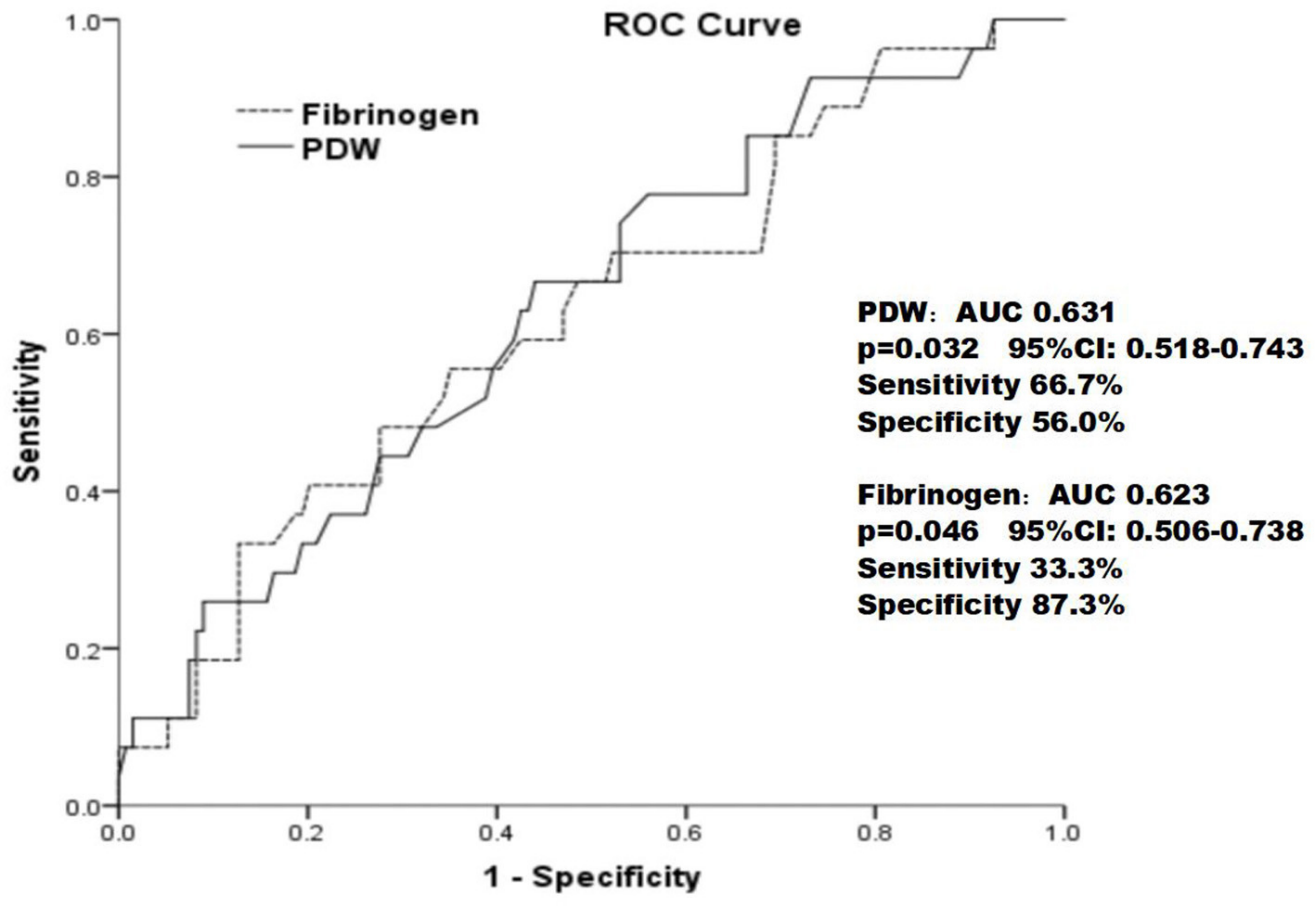
The receiver operating characteristic curve of the PDW and fibrinogen in predicting in-stent restenosis in patients with type 2 diabetes mellitus who underwent drug-eluting stent implantation.

As shown in Figure 3, the ROC curves showed that the PDW cutoff value for predicting ISR rate was 13.15 % with sensitivity of 66.7% and specificity of 56.0% [area under the curve (AUC) = 0.631; 95% CI: 0.518–0.743; *p* = 0.046] and fibrinogen cutoff value for predicting ISR rate was 333.5 mg/dl with sensitivity of 33.3% and specificity of 87.3% (AUC = 0.623; 95% CI: 0.506–0.738; *p* = 0.032).

Based on the threshold for the PDW calculated by Youden's index, the cohort was divided into the two groups: the higher PDW group (PDW ≥ 13.15%, *n* = 77) and the lower PDW group (PDW < 13.15%, *n* = 84).

Based on the threshold for the fibrinogen calculated by Youden's index, the cohort was divided into the two groups: the higher fibrinogen group (fibrinogen ≥ 333.5 mg/dl, *n* = 26) and the lower fibrinogen group (fibrinogen < 333.5 mg/dl, *n* = 135).

**Figure 4:**
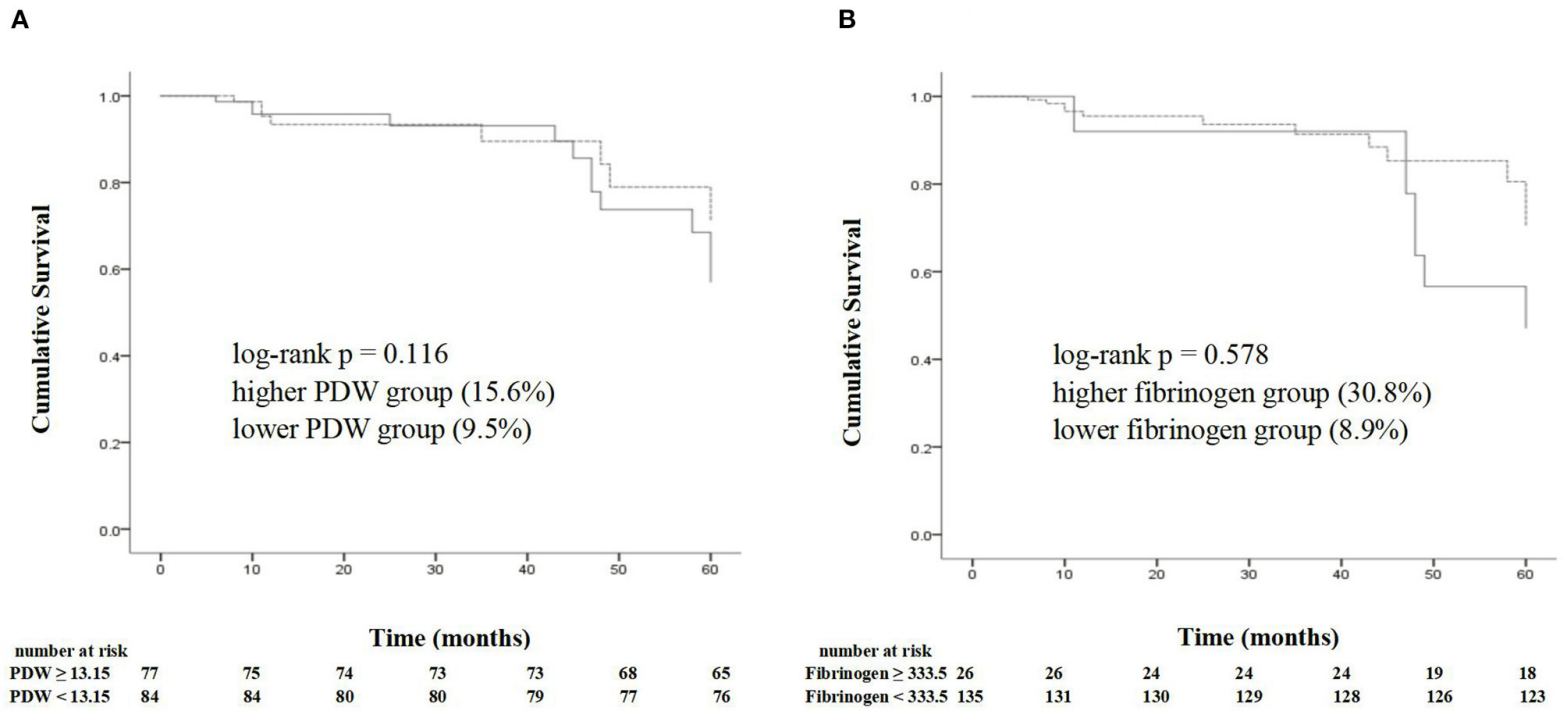
The Kaplan–Meier analysis shows the cumulative survival of target lesion failure according to the cutoff points of a PDW of 13.15% **(A)** and a fibrinogen of 333.5 mg/dl **(B)**. PDW, platelet distribution width.

Of 161 patients, 20 suffered from TLF during 5 years follow-up. The Kaplan–Meier estimate for TLF showed no significant differences in the lower PDW and fibrinogen groups compared with the higher PDW and fibrinogen groups (TLF, 15.6 vs. 9.5%, *p* = 0.578; 30.8 vs. 8.9%, *p* = 0.116, respectively) (Figure 4).

## Discussion

Recent studies have shown that ISR is an independent risk factor for mortality after PCI (23). In this study, we observed that the levels of PDW and fibrinogen were independent risk factors for patients with ISR with stable angina pectoris and T2DM after DES implantation.

First, vascular endothelium induces such overreactions as plaque rupture, platelet, and white blood cell activation when suffered from a mechanical injury after stent implantation, which can induce the release and activity of inflammatory mediators and chemokines, and increase the incidence of ISR and adverse cardiovascular events after PCI (24). Platelets and fibrinogen played an important role in the development of ISR after PCI (25).

Fuster et al. (26) found that mural thrombus occurred in the lumen wall of coronary vessels after PCI, which promoted the occurrence of ISR. Several studies have shown that PDW was strongly associated with major adverse cardiovascular events (MACEs) in patients undergoing PCI. This study showed that the level of PDW was an independent predictor of adverse outcomes in patients with acute coronary syndrome (ACS) hospitalization and postoperative long term (27). Studies have shown that PDW level is an independent predictor of successful thrombolysis in patients with ST-elevation myocardial infarction (STMEI) and the level of PDW in patients with STEMI with failed thrombolysis was significantly higher than that in the successful group (17.7 ± 1.0 vs. 16.4 ± 2.1 fl, *P* < 0.001) (28). Of course, further and larger studies are needed to demonstrate the relationship between PDW and ISR, especially in different patient populations.

Prospective epidemiological studies (3–6) and meta-analyses (29–31) clearly indicate that fibrinogen was an independent risk factor for adverse cardiovascular events, similar in incidence to other major risk factors such as cholesterol and smoking (3–6). In addition, studies have reported an association between fibrinogen and the presence and severity of ISR, suggesting its potential role in the progression of coronary atherosclerosis (7, 8, 32). Fibrinogen is the precursor of fibrin, which is involved in the process of inflammation and thrombosis. Fibrinogen and its metabolites may stimulate endothelial cell deterioration and associated tissue disorders, and increase the release of endothelial cell-derived growth factors, leading to endothelial dysfunction. In addition, fibrinogen may stimulate the growth of smooth muscle cells, leading to ISR. Some studies have shown that fibrinogen is closely related to the prognosis and severity of coronary artery disease (33, 34). Platelet deposition and thrombosis are the basic mechanisms leading to ISR. The relationship between fibrinogen levels and ISR and the pathophysiological explanation of ISR was a controversial issue. There were also some studies that have found no significant association between fibrinogen levels and ISR, but these studies all have some limitations, such as the limitation of sample size and other factors that may affect the final results (35, 36).

The relationship between plasma fibrinogen levels and ISR in patients with stable coronary artery disease treated with PCI was described in these studies (37, 38). According to these studies, ISR was mediated by the interaction between fibrinogen and platelets. This study showed that plasma fibrinogen levels were higher in patients with ACS and plasma fibrinogen levels ≥ 350 mg/dl in patients with ACS are independent predictors of MACEs (39). Recent studies have shown that patients with ACS with higher fibrinogen levels (>417 mg/dl) compared to lower fibrinogen levels (≤417 mg/dl) have higher in-hospital mortality and that the incidence and severity of ISR in patients with STEMI after PCI were positively correlated with high fibrinogen levels (40).

Therefore, this study focused on patients with stable coronary heart disease and T2DM undergoing DES implantation and our results showed that high plasma fibrinogen levels and higher PDW levels were also positively associated with ISR. But PDW and fibrinogen levels as the predictive values were not strongly to predict the occurrence ISR. In the final, we can obtain that high plasma fibrinogen levels and high PDW levels had no predictive value for the occurrence of TLF in the Kaplan–Meier curves.

### Strengths and Limitations of This Study

Although there were many studies about that plasma levels of PDW and fibrinogen predict the occurrence of ISR after DES implantation in patients with coronary heart diseases, the vast majority of studies have enrolled patients with ACS or myocardial infarction. Perhaps considering the poor prognosis of these patients, the high probability of ISR is higher, which requires early intervention and prediction. Nevertheless, patients with stable angina are still common and present in the real world, and the prediction of the prognosis of patients with stable angina after DES implantation is also very necessary. Whether plasma levels of PDW and fibrinogen were predictive values on the occurrence of ISR after DES implantation in patients with stable angina pectoris is still unknown. Therefore, this study included patients with stable angina pectoris and T2DM, which was advanced and innovative.

There are some limitations that can affect these results; first, this study was retrospective, with patients retrospectively reviewing coronary artery angiography due to recurrent chest pain, but there were many patients who had <50% of the in-stent area but no clinical symptoms and may not have been reviewed for coronary artery angiography, which affected the ultimate outcome. In addition, all the patients included in this study were patients with stable angina pectoris with T2DM. During the follow-up period, poor blood glucose control may affect ISR. Finally, all the patients who underwent coronary artery angiography without intravascular imaging or functional intervention techniques may affect the accuracy of results.

## Conclusion

Higher levels of PDW and fibrinogen were associated with the incidence of ISR in patients with stable angina with T2DM after DES implantation, but were not independent predictors of TLF.

## Data Availability Statement

The original contributions presented in the study are included in the article/supplementary material, further inquiries can be directed to the corresponding author.

## Ethics Statement

Written informed consent was obtained from the individual(s) for the publication of any potentially identifiable images or data included in this article.

## Author Contributions

DC and XY designed the research, statistical analysis, led the interpretation of research findings, and revised the manuscript. DC, XY, AW, and SL participated in design, data collection, dataset generation, statistical analysis, and drafting of the manuscript. YD and JZ participated in dataset generation, statistical analysis correction, language correction, and drafting of the manuscript. All authors have read and approved the final manuscript.

## Conflict of Interest

The authors declare that the research was conducted in the absence of any commercial or financial relationships that could be construed as a potential conflict of interest.

## Publisher's Note

All claims expressed in this article are solely those of the authors and do not necessarily represent those of their affiliated organizations, or those of the publisher, the editors and the reviewers. Any product that may be evaluated in this article, or claim that may be made by its manufacturer, is not guaranteed or endorsed by the publisher.
